# Transcutaneous bilirubin monitoring predicts unexplained late-onset hemolysis in a very low birthweight infant

**DOI:** 10.1186/s13104-016-1970-1

**Published:** 2016-03-10

**Authors:** Miwako Nagasaka, Tomoe Kikuma, Sota Iwatani, Daisuke Kurokawa, Keiji Yamana, Kaori Maeyama, Tsubasa Koda, Hisayuki Matsumoto, Mariko Taniguchi-Ikeda, Kazumoto Iijima, Hajime Nakamura, Ichiro Morioka

**Affiliations:** Department of Pediatrics, Kobe University Graduate School of Medicine, 7-5-2, Kusunoki-cho, Chuo-ku, Kobe, 650-0017 Japan; Clinical Laboratory, Kobe University Hospital, Kobe, Japan

**Keywords:** Hemolysis, Total serum bilirubin, Transcutaneous bilirubin, Very low birthweight infant

## Abstract

**Background:**

In term infants, transcutaneous bilirubin (TcB) monitoring can be used to predict hemolytic hyperbilirubinemia. However, it is not clear whether the technique can also be used to predict unexplained late-onset hemolysis in very low birthweight (VLBW) infants.

**Case presentation:**

The case was an infant with a birthweight of 1154 g who developed unexplained late-onset hemolysis at 8 days of age. The hyperbilirubinemia rapidly worsened, and therefore both phototherapy and exchange transfusion were performed. TcB levels were measured using the JM-105 jaundice meter and found to have increased by >3 mg/dL since before the onset, demonstrating for the first time that the device clearly detects changes in hemolytic rate.

**Conclusions:**

Although TcB levels did not correspond directly with total serum bilirubin levels in VLBW infants, the two values exhibited parallel changes in this case. Therefore, serial TcB monitoring may be useful in the early prediction of unexplained late-onset hemolysis in VLBW infants.

## Background

Several cases have been reported of hemolytic episodes occurring unexpectedly in newborn infants, especially very low birthweight (VLBW) infants, who were within 2–3 weeks old and had a stable cardiovascular status [1–4]. This condition is known as late-onset hemolysis, and it can develop severe hemolytic hyperbilirubinemia. Despite this, its cause is unknown and its onset is difficult to predict; the resulting delayed diagnosis leads to a risk of kernicterus.

Daily blood sampling to measure total serum bilirubin (TSB) may allow early prediction of late-onset hemolysis; in practice however, this is not a suitable option for VLBW infants. Herein, we report the first case of a VLBW infant with unexplained late-onset hemolysis that was predicted using the JM-105 jaundice meter (Konica Minolta, Inc., Tokyo, Japan), which allows serial monitoring of transcutaneous bilirubin (TcB) on the basis of yellowness below the skin.

## Case presentation

A Japanese girl infant weighing 1154 g was born at 29 weeks and 6 days of gestational age; her mother had received a tocolytic agent and betamethasone due to a risk of premature delivery. However, this treatment had failed to inhibit uterine contractions, and the girl infant was delivered by emergency caesarean section. The girl’s Apgar scores were four and six at one and 5 min after birth, respectively. The newborn was diagnosed with respiratory distress syndrome, so her trachea was intubated and an artificial pulmonary surfactant was administered. When the girl was 1 day old, she was extubated; her respiratory condition was stable at this time and she was further treated using nasal directional positive airway pressure (DPAP), as well as a xanthine derivative. Her arterial duct closed spontaneously at around this time. When she was 2 days old, elevated TSB levels were found (9.6 mg/dL), and she was immediately treated using conventional phototherapy, which continued until she was 5 days old. From the time she was 2 days old, the girl was tube-fed; her clinical course was favorable until the event described below.

When the girl was 7 days old, her percutaneous oxygen saturation (SpO_2_) decreased to around 90 %, and residual milk formed; furthermore, blood methemoglobin was elevated at 2.4 %. A day later, when she was 8 days old, TcB levels in the back were found to be elevated to 10 mg/dL, and a blood test revealed that both TSB and unbound bilirubin (UB) were elevated (to 10.8 mg/dL and 0.42 µg/dL, respectively). For these reasons, conventional phototherapy was resumed (phototherapy or exchange transfusion is initiated when TSB or UB measured by FDA-approved UB analyzer [Arrows, Osaka, Japan] reached the following cut-offs in a Japanese guideline: TSB 12 mg/dL or UB 0.3 µg/dL for phototherapy and TSB 18 mg/dL or UB 0.8 µg/dL for exchange transfusion) [5]. When the girl was 9 days old, cyanosis was noted, and the SpO_2_ had decreased to 83 % (nasal DPAP parameters: positive end expiratory pressure = 5 cmH_2_O, fraction of inspired oxygen = 0.4). Nonetheless, she was doing well, with stable vital signs; her heart rate was 140 beats/min, and her blood pressure was 65/39 mmHg. Subsequently, anemia became markedly worse (hemoglobin levels = 7.8 g/dL), and blood carboxyhemoglobin (COHb) increased to 5 %. Although conventional phototherapy was administered, hyperbilirubinemia also worsened (TSB = 18.4 mg/dL, UB = 1.48 µg/dL). Because of this rapid deterioration in terms of both anemia and hyperbilirubinemia, treatment was promptly switched to intensive phototherapy, and an exchange transfusion was performed. Thereafter, SpO_2_ immediately increased to 100 %, and cyanosis subsided. However, as the TSB and UB levels remained at 15.9 mg/dL and 1.15 µg/dL, respectively, a second exchange transfusion was performed. When she was 11 days old, as the TSB and UB levels had decreased, treatment was switched back to conventional phototherapy; this therapy was completed when the patient was 13 days old; at that time, the TSB and UB had decreased to 5.2 mg/dL and 0.14 µg/dL, respectively. No hemolytic episodes occurred thereafter (TcB = 3.8–8.5 mg/dL, TSB = 4.9–7.5 mg/dL). The results of both magnetic resonance imaging and the auditory brain stem response test were normal at the time of discharge (38 weeks of corrected gestational age). At the corrected age of 18 months, the girl could walk without assistance and speak some words; she showed no symptoms of kernicterus.

**Fig. 1:**
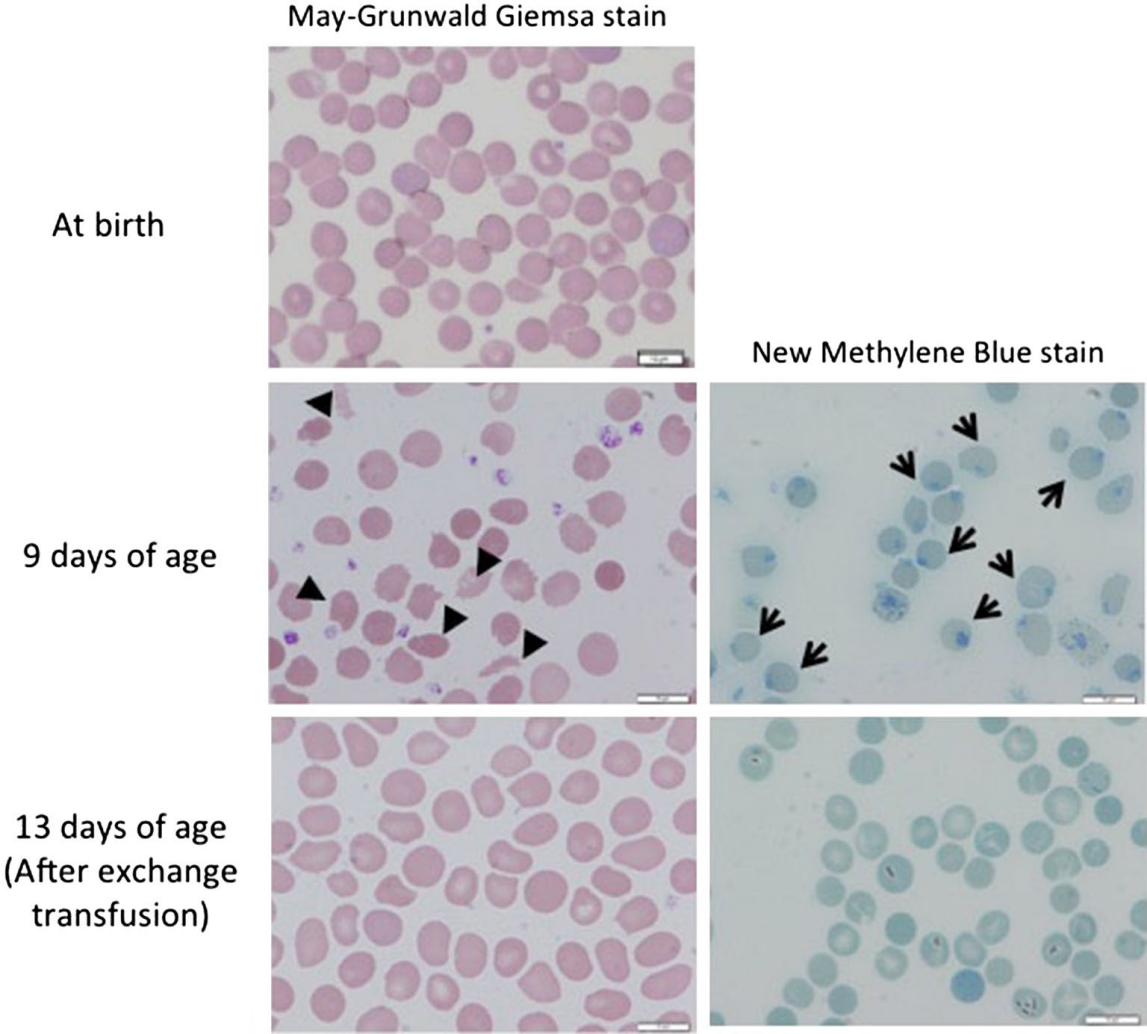
Peripheral blood pictures. Schistocytes (indicated by *arrowheads*) and Heinz bodies in the erythrocytes (indicated by *arrows*) can be seen during the development of hemolytic hyperbilirubinemia

As for the cause of hemolytic jaundice, neither blood group incompatibility (mother and child blood groups: type O and Rhesus D positive, respectively) nor irregular antibodies (anti-C, c, D, E, e, Di^a^, Di^b^, Fy^a^, Fy^b^, Jk^a^, Jk^b^, S, s, M, N, P1, Le^a^, and Le^b^ antibodies) were detected. Glucose-6-phosphate dehydrogenase deficiency is extremely rare in the Japanese population [6, 7]. Although most hemolytic cases due to glucose-6-phosphate dehydrogenase deficiency in Japan are boys from a mother with non-Japanese ethnicity [6, 7], this present case is a girl and her parents were Japanese without the history of hemolysis. No hemolytic relapses occurred thereafter in this case. A peripheral blood picture taken when the girl was 9 days old confirmed schistocytes and Heinz bodies (Fig. 1). Considering her clinical course, the infant was diagnosed with unexplained late-onset hemolysis.

## Transition of TcB

**Fig. 2:**
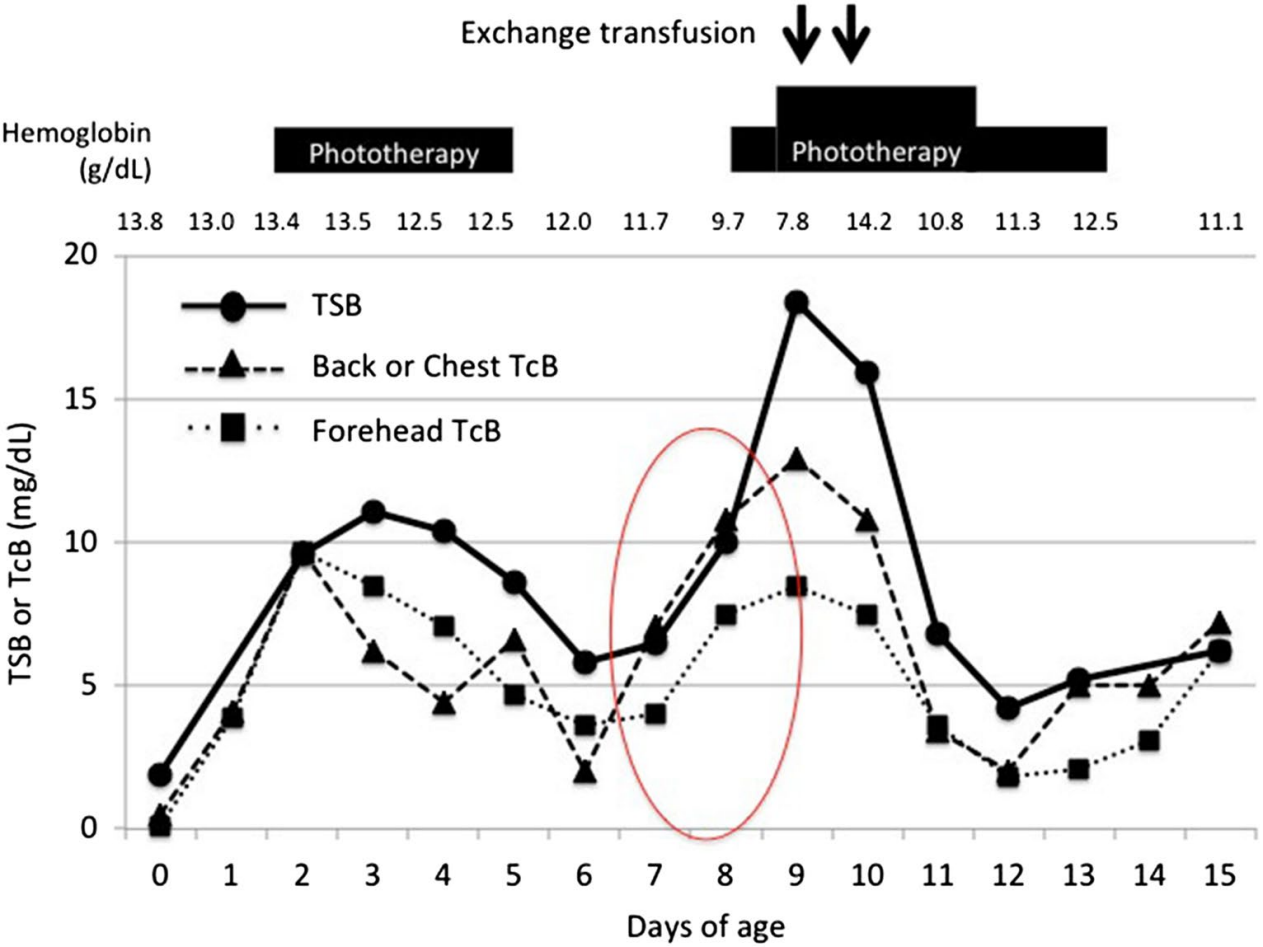
Transition of TSB and TcB levels. When a hemolytic episode occurred at 8 days of age, the TcB level increased by >3 mg/dL compared to that at 7 days of age. *TcB* transcutaneous bilirubin, *TSB* total serum bilirubin

Figure 2 shows the changes in TSB and TcB levels over time, the latter of which was measured using the JM-105 jaundice meter. TcB levels varied in parallel with TSB levels. The linear regression analysis between TSB and forehead TcB levels, or back or chest TcB levels in this present case showed the following results: the regression equation and coefficient of determination were y = 0.52x + 0.84 and 0.64 for forehead TcB levels or y = 0.69x + 0.37 and 0.68 for back or chest TcB levels.

When the girl was 8 days old, the TcB level was found to have increased by >3 mg/dL over that found when the girl was 7 days old. Specifically, TcB levels in her back rose from 7.1 mg/dL at 7 days of age to 10.8 mg/dL at 8 days of age; those in the forehead rose from 4.0 mg/dL at 7 days of age to 7.5 mg/dL at 8 days of age. Further evaluation indicated that this was due to an acute hemolytic episode.

## Discussion

To our knowledge, this is the first report describing the prediction of unexplained late-onset hemolysis using TcB monitoring in a VLBW infant. The following characteristics are seen in unexplained late-onset hemolysis: (1) the infant is born preterm, especially with a VLBW; (2) despite a stable general condition, hemolytic hyperbilirubinemia occurs suddenly 2–3 weeks after birth; (3) SpO_2_ decreases as a result of methemoglobinemia, and this is followed by elevation of blood COHb; (4) the peripheral blood picture shows Heinz bodies; (5) the hemolytic attack is transient and does not relapse; and (6) the condition may develop into severe hyperbilirubinemia, which requires exchange transfusion in addition to phototherapy [3, 4]. The present case showed all these characteristics, allowing a diagnosis of unexplained late-onset hemolysis. The etiology of the condition remains uncertain. One possible causative factor in this disease is oxidative stress, perhaps caused by a combination of the immature antioxidant capacity of the erythrocyte and exposure to highly concentrated oxygen and drugs or toxic agents [3, 8]. Although neonatal Heinz body hemolytic anemia can be induced by exposures of vitamin C [9], phenolic disinfectant [10], toluidine blue [11], or methylene blue [12], the present case did not expose to these agents. Intravenous vitamin K (including sodium hydroxide, lecithin, sorbitol, taurine and glycerin) and xanthine derivative (including ethylenediamine), and oral Daikenchuto were taken before appearing hemolysis. There was no exposure to cleansers on her skin.

As this disease causes severe hyperbilirubinemia, the infant is at risk of developing kernicterus. Therefore, it is important that hemolytic hyperbilirubinemia is predicted early and that treatment is initiated likewise. An antecedent decrease in SpO_2_, as well as methemoglobinemia, can be considered indicators of the disease. However, as these phenomena are non-specific, it is difficult to predict only hemolysis on their basis alone. Instead, measuring TSB daily may allow more specific prediction. Unfortunately, this is an ethically unsuitable option in routine clinical practice, because such measurement requires frequent blood sampling, thereby increasing stress on the infant, as well as accelerating the deterioration of anemia. Thus, the non-invasive measurement of TcB may be a useful tool for predicting this disease.

In full-term and late-preterm infants, TcB measurements are used as a non-invasive tool for predicting hemolytic hyperbilirubinemia such as that caused by blood group incompatibility as well as hyperbilirubinemia from other causes [13–15]. On the other hand, it remains unclear whether TcB measurement can be used to predict unexplained late-onset hemolysis in VLBW infants. In the present case, the TcB level increased by >3 mg/dL from 7 days of age to eight, and these changes in TcB levels were clearly confirmed. Thus, the infant was immediately treated using phototherapy, and subsequently using exchange transfusion.

In this present case, a diagnosis of hemolysis was made based on a drop in hemoglobin level, detection of Heinz bodies in the erythrocytes, and elevated COHb. Measurements of end-tidal breath carbon monoxide corrected for ambient carbon monoxide (ETCOc) are used as an index of hemolysis [16]. If serial ETCOc measurements can be applied to preterm or VLBW infants, ETCOc may be a useful tool for early detection of unexplained late-onset hemolysis.

## Conclusion

Serial TcB monitoring may be a useful tool for predicting hemolysis early, not only in term, but also in preterm VLBW infants.

## Ethics

Clinical data collection was approved by the Ethical Committee of Kobe University Graduate School of Medicine (no. 1450).

## Consent

Written informed consent was obtained from the parents of the patient to publish this report.

## Abbreviations

COHb: carboxyhemoglobin
DPAP: directional positive airway pressure
ETCOc: end-tidal breath carbon monoxide corrected for ambient carbon monoxide
SpO_2_: percutaneous oxygen saturation
TcB: transcutaneous bilirubin
TSB: total serum bilirubin
UB: unbound bilirubin
VLBW: very low birthweight
